# Is PTSD symptomatology a mechanism of emotional regulation? Insights from an interdisciplinary point of view

**DOI:** 10.3389/fpsyg.2024.1454900

**Published:** 2025-01-17

**Authors:** Juan Pablo Rojas-Saffie, Nicolás Álamos-Vial, Manuela Pinzón-Merchán

**Affiliations:** ^1^Department of Psychology, Faculty of Education, Psychology and Family, Universidad Finis Terrae, Santiago, Chile; ^2^Center for Research in Education, Psychology and Family (CIPEF), Faculty of Education, Psychology and Family, Universidad Finis Terrae, Santiago, Chile

**Keywords:** trauma, psychopathology, PTSD, self-regulation, emotional dysregulation, Thomistic anthropology, Integral psychology of the person, Thomistic psychology

## Abstract

Post-traumatic stress disorder (PTSD) symptomatology has historically been considered a psychic ailment that is part of a mental disorder. However, it has often been proposed that it could play an adaptive role, in that it would prevent individuals from being exposed to content or situations that they would not be prepared to process. Within the literature on emotional regulation (ER), PTSD symptomatology has commonly been linked to the concept of emotional dysregulation (ED). However, some definitions open the possibility that traumatic symptomatology could be considered ER, from which delicate conclusions would follow. To resolve this dilemma, we turn to interdisciplinary dialogue, specifically with the aid of Thomistic anthropology, whose concepts allow us to understand precisely the relationship between voluntary and involuntary processes and the close relationship between the concepts of reason and regulation. Even though part of the PTSD symptomatology involves psychic mechanisms aimed at modifying emotional states, it is concluded that it is necessary to continue conceptualizing it as ED. The theoretical and practical implications of this discussion are reviewed.

## 1 Introduction

The symptomatology of Post-traumatic stress disorder (PTSD) plays a leading role in the origin of clinical psychology. Hundred and thirty years ago, Breuer and Freud (1885/1936) postulated that psychic trauma could trigger a particular type of hysteria, traumatic hysteria, which was characterized, among other things, by a set of spontaneous psychosomatic phenomena, that are automatic and uncontrolled by the subject. Freud (1917/1977, XVI) departed early from the psychiatric approach of his time, which considered neurotic symptomatology as the simple effect of an imbalance of the nervous system, stating that it should be interpreted according to a psychological sense; somehow, the symptoms fulfilled a function within the psychic organization of his patients. In particular, the forgetting of traumatic events would prevent the patient from facing the irreconcilability of such contents with the “ethical and aesthetic purposes of the ego” (Freud, 1917/1977, XVI, p. 151). Psychoanalytic therapy proposed that unraveling the meaning of the symptom was fundamental for patients to recover psychic health.

The symptomatology of PTSD has been linked repeatedly to the concept of emotional dysregulation (ED) (Carmassi et al., 2022; Powers et al., 2015a; Raudales et al., 2019). However, some authors have glimpsed an adaptive role in some PTSD symptoms. For example, forgetting some memories could be a mechanism to prevent the self from dealing with content that it is unable to cope with Modai (1994). Freud himself (Freud, 1917/1977) claimed that neurotic symptoms could be perpetuated over time due to their secondary gains. From this point of view, PTSD symptomatology could be reread in the key of emotional regulation (ER). However, such a reading is complicated, since ER and ED have been considered as incompatible phenomena: “The failure to regulate emotion is called dysregulation” (Macklem, 2008, p. 13).

The present study will attempt to explore whether PTSD symptomatology can be really understood as an ER, or whether it is actually more appropriate to keep it within the category of ED. The relevance of this answer is evident from a theoretical perspective. If PTSD symptomatology involves some ER mechanism, then the extent to which PTSD is properly a pathology would have to be questioned. Furthermore, the difference between ER and ED would become much more blurred, making this distinction less relevant. However, these questions could also have practical implications. If PTSD symptomatology involves ER mechanisms and has some adaptive value, to what extent should this symptomatology be tolerated or even incorporated into normal life? Would it be appropriate for the clinician to aim to eliminate it? Considering that traumatic experiences are a recurrent theme in people’s lives and in psychological consultation, the answer to this question is particularly relevant.

The interpretation of PTSD symptomatology from the point of view of ER has been tangentially touched upon in some publications (Ford, 2013; Cole et al., 2019) and also in the clinical literature, especially psychoanalytic literature (Freud, 1915/1961; see also Baumeister et al., 1998; Derakshan et al., 2007). However, the topic does not seem to have been directly addressed before, let alone from an interdisciplinary perspective, which is key to exploring those questions in psychology that are subject to philosophical perspectives. In particular, in this article we will develop a Thomistic approach, which has been shown to be of great use in dialoguing with the dilemmas of contemporary psychology (e.g., Asociación de Psicología Integral de la Persona, 2022; Cartagena, 2021; Cornelius, 2006; Cubillos, 2022; De Haan, 2019; Droste, 2022; Dryden, 2016; Fowers et al., 2023; Marple et al., 2024; Navarini and De Monte, 2019; Ratchford et al., 2023; Rojas-Saffie and García-Matte, 2024; Rojas-Saffie et al., 2024; Schell, 2022; Spalding et al., 2019; Suazo, 2022; Verdier, 2022).

In order to achieve a fruitful interdisciplinary dialogue, we will begin by reviewing the concepts of trauma, PTSD symptomatology, ER and ED in the literature. Then, we will consider some concepts from Thomistic anthropology to distinguish various pathways that enable emotion regulation and to clearly differentiate ER and ED. In the light of this dialogue, we will try to answer whether PTSD symptomatology can be considered ER.

## 2 Theoretical framework

### 2.1 Trauma

The concept of psychological trauma has been the subject of multiple discussions and debates (e.g., Archambeau et al., 2011; Blair et al., 2020; Gradus and Galea, 2022; van der Hart et al., 1989; Isobel et al., 2017; Jongedijk et al., 2023; Nadal et al., 2019; Padykula and Conklin, 2010; Sar and Ozturk, 2006; Tennant, 2004; Weathers and Keane, 2007). Innumerable definitions of trauma have been proposed, which has led to difficulty in achieving cross-sectional consensus (Gershuny, 1999; Weathers and Keane, 2007).

Pierre Janet was one of the first authors to describe what we now call Post-Traumatic Stress Disorder (van der Kolk, 2000b). According to the French psychiatrist, memory encoding and retrieval are fundamental to the functioning of the mind. This is because, throughout life, individuals would organize and integrate their memories and personal experiences, allowing them to develop flexible cognitive schemas. This would enable them to manage future challenges and appropriate actions in the present. He observed that hysterical patients, often traumatized, lacked the ability to integrate traumatic memories, hindering their ability to assimilate new experiences (van der Kolk et al., 1994).

According to this approach, the memory of trauma would persist as “unconscious fixed ideas that cannot be “liquidated” as long as they have not been translated into a personal narrative and instead continue to intrude as terrifying perceptions, obsessional preoccupations and somatic re-experiences such as anxiety reactions” (van der Kolk et al., 1994, p. 585). In the words of Balaev (2018), “trauma is thus defined in relation to the process of remembering and as an event harbored within the unconscious that causes a splitting of the ego or dissociation” (p. 361).

Nowadays, the concept of trauma is often approached from a more descriptive perspective, as can be seen in PTSD, the DSM-V-TR diagnosis most closely linked to trauma. According to it, trauma occurs when someone is exposed to “actual or threatened death, serious injury, or sexual violence” (American Psychiatric Association, 2022, p. 303), producing a series of symptoms that lead to “clinically significant distress or impairment in social, occupational, or other important areas of functioning” (Id.).

### 2.2 PTSD symptomatology

For the purposes of this article, we will focus exclusively on PTSD symptomatology as described in the DSM-V-TR (American Psychiatric Association, 2022). It is right that there is valuable literature about its symptomatology (e.g., Carmassi et al., 2022; van der Kolk et al., 1994), however, our interest is not to make an exhaustive review, but to take a proposal and examine whether it is possible to analyze it from an ER point of view. The DSM-V-TR proposal has seemed appropriate to us because it is one of the most accepted and well-known.

The diagnosis of PTSD in the DSM-V-TR includes 8 criteria, of which 4 refer directly to symptomatology (criteria B, C, D and E). Criterion B deals with intrusive symptoms; criterion C, with avoidant symptoms; criterion D, with negative alterations in cognition and mood; and criterion E, with alterations in arousal and reactivity. In addition, the DSM-V-TR (American Psychiatric Association, 2022) contemplates the necessity of identifying whether PTSD is accompanied by dissociative symptoms such as depersonalization or derealization (see Table 1).

**Table 1:** Posttraumatic stress disorder diagnostic criteria by DSM-V-TR (American Psychiatric Association, 2022).

Criteria related to symptomatology	Symptoms
B. Presence of one (or more) of the following intrusion symptoms associated with the traumatic event(s), beginning after the traumatic event(s) occurred:	1. Recurrent, involuntary, and intrusive distressing memories of the traumatic event(s). 2. Recurrent distressing dreams in which the content and/or affect of the dream are related to the traumatic event(s). 3. Dissociative reactions (e.g., flashbacks) in which the individual feels or acts as if the traumatic event(s) were recurring. (Such reactions may occur on a continuum, with the most extreme expression being a complete loss of awareness of present surroundings). 4. Intense or prolonged psychological distress at exposure to internal or external cues that symbolize or resemble an aspect of the traumatic event(s). 5. Marked physiological reactions to internal or external cues that symbolize or resemble an aspect of the traumatic event(s).
C. Persistent avoidance of stimuli associated with the traumatic event(s), beginning after the traumatic event(s) occurred, as evidenced by one or both of the following:	1. Avoidance of or efforts to avoid distressing memories, thoughts, or feelings about or closely associated with the traumatic event(s). 2. Avoidance of or efforts to avoid external reminders (people, places, conversations, activities, objects, situations) that arouse distressing memories, thoughts, or feelings about or closely associated with the traumatic event(s).
D. Negative alterations in cognitions and mood associated with the traumatic event(s), beginning or worsening after the traumatic event(s) occurred, as evidenced by two (or more) of the following:	1. Inability to remember an important aspect of the traumatic event(s) (typically due to dissociative amnesia and not to other factors such as head injury, alcohol, or drugs). 2. Persistent and exaggerated negative beliefs or expectations about oneself, others, or the world (e.g., “I am bad,” “No one can be trusted,” “The world is completely dangerous,” “My whole nervous system is permanently ruined”). 3. Persistent, distorted cognitions about the cause or consequences of the traumatic event(s) that lead the individual to blame himself/herself or others. 4. Persistent negative emotional state (e.g., fear, horror, anger, guilt, or shame). 5. Markedly diminished interest or participation in significant activities. 6. Feelings of detachment or estrangement from others. 7. Persistent inability to experience positive emotions (e.g., inability to experience happiness, satisfaction, or loving feelings).
E. Marked alterations in arousal and reactivity associated with the traumatic event(s), beginning or worsening after the traumatic event(s) occurred, as evidenced by two (or more) of the following:	1. Irritable behavior and angry outbursts (with little or no provocation) typically expressed as verbal or physical aggression toward people or objects. 2. Reckless or self-destructive behavior. 3. Hypervigilance. 4. Exaggerated startle response. 5. Problems with concentration. 6. Sleep disturbance (e.g., difficulty falling or staying asleep or restless sleep).
Specify whether:	With dissociative symptoms: 1-Depersonalization: Persistent or recurrent experiences of feeling detached from, and as if one were an outside observer of, one’s mental processes or body (e.g., feeling as though one were in a dream; feeling a sense of unreality of self or body or of time moving slowly). 2-Derealization: Persistent or recurrent experiences of unreality of surroundings (e.g., the world around the individual is experienced as unreal, dreamlike, distant, or distorted).

### 2.3 Emotional regulation

As with *trauma*, the concept of ER does not have a consensus definition either. This has led to the use of the same term to designate similar but different realities (Rojas-Saffie and García-Matte, 2024). Emotion regulation is concerned with modulation or maintaining of feelings, behaviors, and physiological responses that comprise an emotion (Gross, 2002). For the purposes of this article, we will initially use as operational definition the one proposed by Gross (2015): “the defining feature of emotion regulation is the activation of a goal to influence the emotion trajectory” (p. 5).

The need to recognize that some inadvertent processes can be considered ER is manifested in the distinction between explicit and implicit ER (Dillon et al., 2007; Koole et al., 2015; Sperduti et al., 2017; Tupak et al., 2014). Explicit ER is that which occurs from conscious processes, with some level of control, and associated with some insight (Gross and Thompson, 2007; Gyurak et al., 2011; Phillips et al., 2008). On the other hand, implicit ER may be defined as “any process that operates without the need for conscious supervision or explicit intentions, and which is aimed at modifying the quality, intensity, or duration of an emotional response” (Koole and Rothermund, 2011). Implicit ER can thus be instigated even when people do not realize that they are engaging in any form of emotion regulation and when people have no conscious intention of regulating their emotions (Braunstein et al., 2017; Gyurak et al., 2011; Torre and Lieberman, 2018).

Although implicit ER is presumably unconscious, that does not mean that it lacks a goal-directed nature. For instance, Hopp et al. (2011) propose that “despite this variety of unconscious emotion-regulation processes, theoretical considerations suggest that one common pathway may underlie many of them: unconscious goal pursuit, or, implicitly represented values regarding emotion regulation” (p. 533). As Custers and Aarts (2010) explain “unconscious goal pursuit results from the design and workings of the brain and mind, which process and represent behaviorally relevant information in such a way that goal pursuit can be controlled by the social situation without conscious awareness of the activation and operation of the goal” (p. 50). This fits well with approaches that distinguish between conscious and unconscious goal pursuit (Bargh et al., 2001; Bargh and Williams, 2006).

It is important to note that although implicit ER would operate unconsciously, it is not necessarily associated with maladaptation, since “implicit emotion-regulation processes are associated with psychological health outcomes only among individuals who habitually use an adaptive, relatively conscious emotion-regulation strategy” (Hopp et al., 2011, p. 541).

### 2.4 Emotional dysregulation

Emotion dysregulation (ED) can be defined as “patterns of emotional experience or expression that interfere with appropriate goal-directed activity” (Thompson, 2019, p. 806), suggesting that one of its core elements is the opposition between goal-directed activity and emotional activity. Other approaches have emphasized its maladaptive nature (e.g., Beauchaine, 2015), noting that ED interferes with appropriate behaviors, or involves emotions expressed or experienced inappropriately for the context (Cole et al., 2019). According to D’Agostino et al. (2017) emotional dysregulation has five dimensions: decreased emotional awareness, inadequate emotional reactivity, intense experience and expression of emotions, emotional rigidity and cognitive reappraisal difficulty.

ED has been identified as a key factor in the etiology and maintenance of multiple psychopathologies (Gasol et al., 2022; Keeshin et al., 2021; Pop-Jordanova, 2023) such as depression (Gao et al., 2022), anxiety (Suveg et al., 2010), sleep disturbance (Zhou et al., 2023), borderline personality disorder (Linehan, 1993; Ford and Courtois, 2014) and attention deficit hyperactivity disorder (Bemmouna and Weiner, 2023).

Many articles on emotional dysregulation implicitly refer to it as contrary to emotional regulation. However, although in the long term it represents a risk, it has been observed that emotional dysregulation involves patterns of behavior that may be adaptive in the immediate context, as a means of survival for the person (Cole et al., 2019; Webermann and Murphy, 2018). In that sense, emotional regulation and emotional dysregulation would not be quite opposites. In the words of Cole et al. (2019): “emotion dysregulation is a pattern of emotion regulation that is interpreted as dysregulated based on the implications for interfering with healthy, competent development” (p. 1192). Similarly, Linehan (1993) proposes that emotional dysregulation is at the basis of behaviors such as deliberate self-harm; however, at the same time, she conceptualizes it as an emotional regulation strategy (see Briere and Gil, 1998; Gratz and Roemer, 2003).

### 2.5 Relationship between PTSD and ER/ED in the literature

Studies have proposed two types of relationship between PTSD and ER. The first of these we will call the “inverse relationship,” in the sense that the occurrence of one of the variables prevents the occurrence of the other. And the second one, a “direct relationship” between PTSD symptomatology and ER, in the sense that they are mutually favoring variables.

#### 2.5.1 The inverse relationship

A recurrent conclusion in the literature postulates that PTSD generates difficulties in the ER (Ehring and Quack, 2010; Ion et al., 2023; van der Kolk, 2007a; Powers et al., 2015b; Yin et al., 2022). As mentioned by Ehring and Quack (2010), “a number of authors suggest that emotion regulation difficulties are one of the complex symptoms that specifically develop after early-onset chronic interpersonal trauma” (p. 588; see also Lee et al., 2018). Southam-Gerow and Kendall (2002) found that children who have suffered abuse are more likely to have difficulties in emotional expression, recognition and communication, which influence emotional regulation. Another study found that abused children have greater problems in emotional regulation and behavioral problems such as the presence of anger, less self-control, and greater degrees of negative emotions (Erickson et al., 1989). In addition, it has been found that emotion regulation problems persist into adulthood and may be an important mechanism in which childhood abuse leads to adult psychopathology (Alink et al., 2009; Kim and Cicchetti, 2010). Trauma has been taken by some authors as a source of mental disorders that could be related to the lack of ER such as, for example, adolescent behavioral problems and addictions (Perry and Szalavitz, 2007). Ion et al. (2023) found that childhood trauma is associated with reduced emotion regulation success. For their part, Cavicchioli et al. (2021) hold that dissociation, a phenomenon inherently related to trauma, is a maladaptive regulatory mechanism.

Some researchers have shown that ER is learned in the interaction with primary caregivers, as they model adaptive behaviors and guide the exploration of the emotional world. In this sense, children would present a higher propensity to develop trauma because they are in the early stage of acquiring regulatory strategies and developing their attachment system (Ehring and Quack, 2010; van der Kolk, 2007a; van der Kolk, 2007b; Liotti, 2004).

The development of ER is seen as a protective strategy against clinical disorders associated with trauma: mood disorders (Lee et al., 2018) and depression (Yin et al., 2022). ER is a central issue in positively managing the effects of trauma and neglect (van der Kolk, 2014). In fact, it has been argued that early interventions that focus on emotional regulation prove to be positive for people experiencing traumatic situations (van der Kolk, 2007b; Zhou et al., 2023).

This inverse relationship can also be seen in studies that consider the development of ER as a way of processing the trauma associated with PTSD (Benight et al., 2018; Conti et al., 2023; van der Kolk, 2007a; Lee et al., 2018). As stated by van der Kolk (2000a) emphasizing self-regulatory needs may be an advantageous approach in patients susceptible to react adversely to standard treatment for PTSD. On the other hand, self-regulation could be viewed as a key to processing trauma and its consequences adaptively (Benight et al., 2018).

#### 2.5.2 The direct relationship

Finally, a “direct relationship” between PTSD symptomatology and ER can be postulated, in the sense that they are mutually favoring variables. This relationship could be observed in those studies that consider PTSD symptomatology as a protective form of ER. As mentioned by Ford (2013) “dissociation in the wake of psychological trauma appears to be a compensatory self-protective adaptation to threat, which can be understood as the involuntary substitution of survival-based hypervigilance” (p. 240). From this perspective, some PTSD symptoms would be adaptation-oriented:

- Intense or prolonged psychological distress at exposure to internal or external cues that symbolize or resemble an aspect of the traumatic event(s). (B4): This distress may be a way to dissuade the individual from exposure to life-threatening situations.
- Avoidance of or efforts to avoid distressing memories, thoughts, or feelings about or closely associated with the traumatic event(s) (C1): The avoidance of these cognitions and affects could be aimed at avoiding the subject the displeasure of reconnecting with traumatic contents.
- Avoidance of or efforts to avoid external reminders (people, places, conversations, activities, objects, situations) that arouse distressing memories, thoughts, or feelings about or closely associated with the traumatic event(s) (C2): The same applies as above.
- Inability to remember an important aspect of the traumatic event(s) (typically due to dissociative amnesia and not to other factors such as head injury, alcohol, or drugs). (D1): Forgetfulness or mental lacunae may be intended to protect the subject from contacting contents that he or she is unable to cope with (Hurtado, 2016).
- Persistent negative emotional state (e.g., fear, horror, anger, guilt, or shame). (D4): Fear, terror and anger could be aimed at keeping individuals prepared to react to any threat. Guilt could be aimed at provoking reflection in individuals so that they discover what their responsibility was in what happened and thus not expose themselves again in the future. Shame could be aimed at avoiding social criticism.
- Markedly diminished interest or participation in significant activities (D5): This could be aimed at avoiding exposure to situations where serious and unexpected threats to life could arise.
- Hypervigilance (E3): The fixation of attention on potential risks would help the subject to anticipate, avoiding the emergence of sudden and unforeseen threats.
- Dissociative symptoms: The dissociative mechanisms prevent the subject from connecting with the emotion, thereby preventing him from experiencing psychic pain.

Although both types of relationship are supported in the literature, it does not seem reasonable that they are simultaneously correct. Either PTSD symptomatology is inversely related to emotional regulation, or it is directly related to it. If the relationship is inverse, then it would be inappropriate to say that PTSD symptomatology is a mechanism of emotional regulation. But if the relationship is direct, then it would be appropriate to say so. Let us review how interdisciplinary dialogue can aid us in resolving this dilemma.

## 3 Interdisciplinary dialogue

### 3.1 The *cogitative* and its relationship with the rational and sensitive dimensions of the human soul

Thomas Aquinas, in line with Aristotle, proposed that human beings perform their psychological activity thanks to a set of operative capacities called *faculties* (Aquinas, 1920, I, c. 77). According to the nature of the human soul, some of them belong to the sensitive dimension, such as the capacity to sense -external and internal senses- (1920, I, c. 78, a. 3; a. 4) and to have emotions -sensitive appetite- (1920, I, c. 80; c. 81), and others to the rational dimension, such as the capacity to understand -reason- (1920, I, c. 79) and to want -will or rational appetite- (1920, I, c. 80; c. 82). The former operate according to the sensible characteristics of the world and are common to humans and animals. The latter operate according to the essential aspects of things, and are exclusive to human beings (1920, I, c. 78, a. 1). Table 2 represents synthetically the Thomistic faculties scheme.

**Table 2:** Thomas Aquinas’s scheme of human faculties.

Psychic dimension	Cognitive faculties		Appetitive faculties	
Rational dimension	Reason	Faculty that allows the human being to understand, reason and rule over emotions.	Will or rational appetite	Faculty that allows the human being to tend toward the intangible good and make choices.
Sensitive dimension	Internal senses	Memory: faculty that stores images in terms of lived experiences.	Sensitive appetite	Concupiscible appetite: faculty that tends toward tangible good, insofar as delectable.
		Cogitative: faculty that evaluates images as convenient or harmful.		
		Imagination: faculty that forms the internal image of the external stimulus.		Irascible appetite: faculty that tends toward the tangible good, insofar as arduous.
		Common sense: faculty that integrates the information of the stimuli.		
	External senses	Faculties oriented to sense the external world, such as touch, taste, smell, hearing, and sight.		

Despite this multiplicity of faculties, the human soul is considered an indivisible unity, which is expressed in the fact that these faculties do not operate dispersedly, but in a coordinated way (Aquinas, 1920, I, c. 76, a. 3). In fact, human beings, by their nature, are made to live according to their reason and will and accompanied at the same time by emotions (1920, I-II, c. 4). For this to be possible, it is necessary to admit an intrinsic capacity to rule over their affectivity. This implies, on the one hand, that the sensitive appetite is naturally open to be guided (Aquinas, 1920, I, c. 81, a. 3; Aquinas, 1999, c. 8), and on the other hand, that reason is capable of guiding it (Aquinas, 1920, I-II, c. 17, a.7; Botkin, 1921).

Aquinas proposed that every emotional movement arises from a judgment or evaluation made by an internal sense called *cogitative*, which assesses the stimuli as convenient or harmful to the vital interests of the subject (Aquinas, 1920, I, c. 78, a.4). For example, if it is something desirable, but not yet obtained, then desire arises; once it is obtained desire ceases and delight arises.

**Figure 1:**
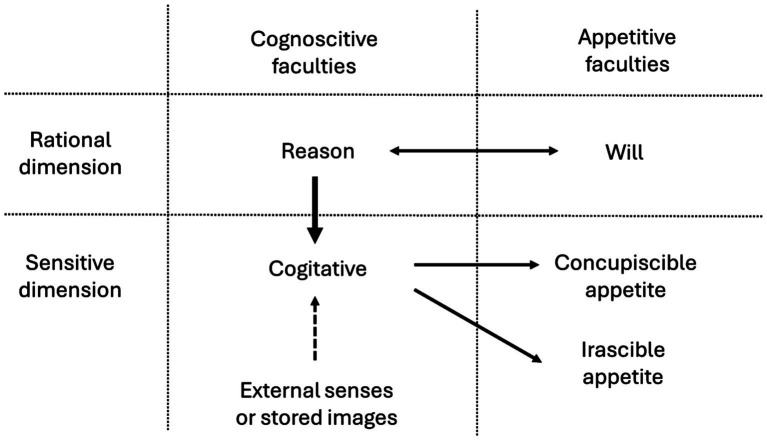
Dynamics of the human psychic faculties. The movements of the concupiscible and irascible appetites are triggered by the valuation of the cogitative. The cogitative is usually moved by reason, but it can also be moved by the external senses or by stored images without the interference of reason. The external senses are moved by external stimuli. The reason and the will move each other mutually.

Now, cogitative can be moved in two ways. First, moved by reason, as, for example, when those who are angry consider the good intentions of others to lessen their anger (Aquinas, 1920, I, c. 81, a. 3). And second, before reason realizes it (Echavarría, 2016), allowing itself to be moved by external or internal stimuli (e.g., memories), as when we pass by a street from our childhood and experience involuntary emotions (see Figure 1).

### 3.2 Voluntary and involuntary emotional modification

As we have already explained, the assessment of the cogitative allows an emotion to arise. Now, we can consider that this emotion can also become a stimulus for the cogitative, moving it to evaluate its convenience or inconvenience. From this second assessment a second emotion will arise, which may compete with the original one and predominate over it, producing an emotional modification.

We have not found Thomistic authors who have described this scenario; however, it can indirectly be deduced from the mechanism proposed by Anna Terruwe to explain psychic repression (Terruwe and Baars, 1981). In the following, we will review two examples of the cogitative elaborating judgments about some emotion, one moved by reason and the other provoked by internal stimuli.

First, let us imagine someone who is afraid of public speaking. Such a fear may arise from a cogitative evaluation similar to this: “it is dangerous to speak in public.” If the person does not have a pathology, he/she will realize that it is an inappropriate affect and could modify it –or at least manage to attenuate it– by means of rational considerations such as: “I have done this before,” “this always happens to me and everything ends up going well,” “this audience is favorable,” “it is normal to feel nervous,” etc. These considerations have the power to move the cogitative to modify its first assessment (“it is dangerous to speak in public”) for another one closer to reason (“speaking in public is possible”), giving rise to a new affective state. The influence of reason on the affections through the cogitative is exemplified by Aquinas (1920): “this can be experienced by each one in himself, since, having recourse to certain general considerations, anger, fear, and other similar passions are mitigated or intensified” (I, c.81, a. 3).

Secondly, let us exemplify when the cogitative is moved by internal stimuli: imagine someone who experiences an erotic desire in conflict with social norms. This emotion is accompanied by pleasant images and memories related to sexual satisfaction, but also by negative ones, such as the pain of their partner and possible social rapprochement. Cogitative may value these latter images as extremely dangerous, causing fear to arise before reason notices it. Now, since fear is a passion that involves avoidance (Aquinas, 1920, I-II, c.42, a.1), it follows that the soul will turn its attention away from the affect perceived as dangerous and also from the cogitative’s original assessment (“this person is desirable”), focusing on other things. Thus, the erotic desire is extinguished and the emotional state changes, without it being necessary to deal with it, because there is no awareness of such desire.

In summary: an emotional modification always implies an assessment of the cogitative about affect. Now, the fact that cogitative can be moved by reason or directly by the senses gives rise to two types of emotional modification (EM), which we will call *voluntary* and *involuntary*. Voluntary EM would occur when the emotional modification comes from the initiative of reason, while involuntary EM would occur when such modification comes from a spontaneous evaluation of the cogitative of an external or internal stimuli (see Table 3). Voluntariness is a dimension present in some definitions of emotional regulation and absent in others, as can be seen in Table 4.

**Table 3:** Types of EM according to their voluntariness.

Type of EM	Faculty that elicits the EM through cogitative	Purpose	Examples
Voluntary	Reason	Ordering the affections to act rationally.	Calming down before presenting in class, reacting wisely to an unforeseen event, etc.
Involuntary	Senses	Diminish displeasure or similar.	Drug cravings, dissociation in response to trauma, nail biting in response to anxiety, etc.

**Table 4:** Examples of definitions related to emotional regulation grouped according to the inclusion of intentional agency.

	**Definition**	**Commentary**
**Definitions that consider intentional agency**	“We define self-regulation as processes by which the self intentionally alters its own responses, including thoughts, emotions, impulses, performance, and behaviors, based on standards” (Baumeister and Vohs, 2016, p. 68).	The intentionality of the self is made explicit, which is considered as the agent executing the ER.
	“We can describe self-regulation as a largely unconscious form of volition that involves, and yet goes beyond, the integrative intelligence of motives. Volitional self-regulation draws not only on those networks of experiences that are relevant for one’s needs but on all autobiographical experiences that have contributed to the development of a coherent self-image” (Kuhl, 2018, p. 544).	It is explicitly mentioned that self-regulation is an act of the will, which does not contradict the fact that it can be unconscious.
**Definitions that do not explicitly state intentional agency**	“Emotion regulation refers to the processes by which individuals influence which emotions they have, when they have them, and how they experience and express these emotions” (Gross, 1998, p. 275).	It is individuals who influence their emotions, but it is not clear whether this is voluntary or not.
	“Emotion regulation may be defined as goal directed processes functioning to influence the intensity, duration and type of emotion experienced” (Gyurak et al., 2011, p. 2).	It is not made explicit who is the agent executing the ER.

### 3.3 Voluntary/involuntary EM and explicit/implicit ER

The concepts of voluntary EM and involuntary EM seem to fit with those of explicit ER and implicit ER. However, although they may overlap in some cases, they do not encompass the same realities, because they arise from different criteria. What defines explicitness of ER is consciousness [i.e., whether the subject is aware of the regulation], whereas what defines voluntariness is wanting [i.e., whether the subject wants to regulate]. Consciousness and wanting, in the Thomistic scheme, are activities of diverse faculties (reason and will respectively), and therefore, they are of a different nature. Indeed, to become conscious is a cognitive act, while to want is an appetitive act (see Figure 1). Consequently, it is not necessary that what is conscious always coincides with what is wanted: there is nothing to prevent the existence of voluntary acts performed unconsciously or inadvertently, and involuntary acts performed consciously.

For Aquinas (1920), an act is voluntary when two conditions are fulfilled (I-II, c. 6, a. 1). First, that the principle of the act be in the agent; in other words, that it be intrinsic. According to this condition, both animals and human beings are capable of acting according to intrinsic principles. The second condition is that there is perfect knowledge of the end, which implies not only knowing for what purpose one wants to perform some act, but also understanding the reason for this end and the proportionality of the act to achieve the end (Aquinas, 1920, I-II, c. 6, a. 2). This means that the agent is able to choose between different means to achieve the end. Only human beings can know the end in this way.

Now, voluntary acts can be conscious, as when one calls a friend to unburden oneself of the problems of the day, or they can be performed unconsciously, as when a person smiles kindly when greeting. The latter act is voluntary in that it is performed by individuals who habitually wish to be kind, and it is unconscious in that kind people are not always aware that they are smiling when they greet. In all these cases there is a voluntary intention to regulate emotionality. Although these are acts with a greater or lesser level of awareness, people always retain self-control. Aquinas (1920) illustrates the compatibility between voluntariness and inadvertence of an act with an example:

One need not always be thinking of the last end, whenever one desires or does something: but the virtue of the first intention, which was in respect of the last end, remains in every desire directed to any object whatever, even though one's thoughts be not actually directed to the last end. Thus while walking along the road one needs not to be thinking of the end at every step. (I-II, c.1, a.6, ad. 3)

The voluntariness of some inadvertent or unconscious acts is well grounded in the thought of Aquinas (1920) through his doctrine of the virtues (I-II, c. 55–67). These habitual dispositions are obtained through conscious and voluntary effort (1920, I-II, c. 63, a. 2). Once acquired, however, the virtues become what Aquinas calls “second nature” (1920, I-II, c. 32, a. 2, ad. 3). This implies that the new spontaneous or automatic reactions will be in accord with the acquired virtue: for example, one who has attained the virtue of good eating-called abstinence-has his appetite regulated so that he spontaneously desires to eat in a balanced way (1920, II-II, c. 146, a. 1). Now, the spontaneity of virtue does not deny the participation of reason; on the contrary, it demonstrates that its powerful influence continues to operate even though the issue is not being consciously considered by the person. This is exemplified by Aquinas (1920) when he states that the virtue of fortitude, which implies the participation of reason (I-II, c. 56, a. 4), is best manifested when a sudden threat arises, which does not allow for premeditation (1920, II-II, c. 123, a. 9).

The novelty of this approach is that there would be no problem in admitting that implicit ER, although unnoticed by the subject, may be under the rule of reason. From this perspective, some of the studies on implicit ER actually deal with voluntary EM. For example, in the *emotional conflict task* (Egner et al., 2008; Etkin et al., 2006), the delay in responding to incongruent stimuli is inadvertent for the participant, but it is reasonable, since such delay allows for better performance. In other words: the delay is unconscious, but it is voluntary, since it is in accordance with the intention to respond well, which can be presupposed in the participants.

On the other hand, there are involuntary acts, which respond to impulses or internal mechanisms that are beyond the control of the will, such as, for example, starting to cry in the middle of a discussion or stuttering when feeling very nervous. In these situations, although the person is aware of his or her actions, he or she is unable to control them. In other words, there are situations in which there is a divergence between the EM that occurs unconsciously and the EM that would have been executed voluntarily if the individual had had the possibility to do so. We can think of addictions: uncontrollable craving is often triggered as a way to regulate negative emotions (Köpetz et al., 2013; Tripp et al., 2015). However, in addiction treatment programs, patients are taught to voluntarily choose more adaptive ways to regulate these emotions. Something similar occurs in some mechanisms that are set in motion in the face of trauma, such as dissociation and the impossibility of remembering (Ford, 2013). We could also consider bodily movements in the face of anxiety, such as nail biting or leg movements (Snorrason et al., 2010).

To understand the nature of involuntary EM, we must consider that, from a Thomistic point of view, human beings have inclinations that derive from their nature (Aquinas, 1920, I-II, c.94, a.2). In the first place, an inclination common to all substances, which consists in the tendency toward the conservation of one’s own being. Secondly, an inclination toward more determined goods, according to what human beings have in common with the other animals, such as sexual union, the education of children, or the establishment in some kind of dwelling. And thirdly, “there is in man an inclination to good, according to the nature of his reason, which nature is proper to him: thus man has a natural inclination to know the truth about God, and to live in society” (Id.).

If the sensitive dimension, through cogitative, is capable of executing acts of EM, it will undoubtedly do so in accordance with the goals that human beings share with the rest of living beings. In other words, involuntary EM would operate in pursuit of goals such as self-preservation, avoidance of displeasure, safety, procreation, breeding, and other interests shared with animals. From this point of view, some PTSD symptoms can be understood as involuntary EM (see Table 5).

**Table 5:** Classification of PTSD symptoms according to the involuntary goal that can be attributed to it.

PTSD symptom	Involuntary pursuit goals
Intense or prolonged psychological distress at exposure to internal or external cues that symbolize or resemble an aspect of the traumatic event(s) (B4).	Self-preservation.
Persistent negative emotional state (e.g., fear, horror, anger, guilt, or shame) (D4).	
Markedly diminished interest or participation in significant activities (D5).	
Hypervigilance (E3).	
Avoidance of or efforts to avoid distressing memories, thoughts, or feelings about or closely associated with the traumatic event(s) (C1).	Avoidance of displeasure.
Avoidance of or efforts to avoid external reminders (people, places, conversations, activities, objects, situations) that arouse distressing memories, thoughts, or feelings about or closely associated with the traumatic event(s) (C2).	
Inability to remember an important aspect of the traumatic event(s) (typically due to dissociative amnesia and not to other factors such as head injury, alcohol, or drugs) (D1).	
Dissociative symptoms.	

### 3.4 Can involuntary emotional modification be considered emotional regulation?

There are at least three arguments that can be put forward to support that involuntary EM can be considered emotional regulation. First, it has already been said that the involuntary operation of the cogitative, from which involuntary EM arises, follows certain rules. Moreover, it acts in accordance with certain ends related to the preservation of the individual. If emotional regulation, as Gyurak et al. (2011) state, “may be defined as goal directed processes functioning to influence the intensity, duration and type of emotion experienced” (p. 2), then nothing would prevent us from considering EM as emotional regulation.

Secondly, we have stated that involuntary EM operates according to what we have in common with other animals, namely, the sensitive dimension. Now, it seems absurd not to admit emotional regulation in animals. A hungry tiger can restrain its desire to pounce on prey until it finds the appropriate moment when such a pounce will bear the expected fruit. Lions that lose the battle to be the alpha male give up their desire to mate with the females. The opossum that is placidly feeding will enter a state that simulates death if a predator appears.

Third, if the symptomatology of PTSD were not emotional regulation, then it would be a chaotic reaction. But as we have just argued, an important part of this symptomatology can be interpreted as an attempt to follow certain rules with adaptive arrangements. This is far removed from the concept of chaos, which implies an absence of discernible patterns. Therefore, it seems more appropriate to consider involuntary EM as emotional regulation.

Despite these arguments, we consider that involuntary EM should not be considered as emotional regulation. To explain our position, we will begin by reviewing the distinction made by Thomas Aquinas between human acts and acts of man. For the Italian thinker, not all actions performed by human beings are properly human:

Of actions done by man those alone are properly called "human," which are proper to man as man. Now man differs from irrational animals in this, that he is master of his actions. Wherefore those actions alone are properly called human, of which man is master. Now man is master of his actions through his reason and will; whence, too, the free-will is defined as "the faculty and will of reason." Therefore those actions are properly called human which proceed from a deliberate will. And if any other actions are found in man, they can be called actions "of a man", but not properly "human" actions, since they are not proper to man as man. (Aquinas, 1920, I-II, c. 1, a. 1)

As we can see, what distinguishes human beings from the rest of the animals is their capacity to reason, which leads them to be masters of their actions. Hence, properly human acts are those that come from his will. On the other hand, the acts that the human being performs without the participation of his will should not be considered human, but acts of the human being. Among these acts, Thomas Aquinas mentions “when one moves one’s foot or hand, or scratches one’s beard” (Aquinas, 1920, I-II, c. 1, a. 1, arg. 3).

From this point of view, only voluntary emotional modification could be considered properly human. Insofar as it is governed by reason and guided by the will, this EM is appropriate to the nature of the human being, which is the rational nature, even if it is executed without warning –as in the example of the kind person who smiles without realizing it–. In contrast, involuntary EM would be a phenomenon that occurs in the human being, an act of the human being, but not a properly human act.

Similarly, if we focus our interest on understanding human ER, then we can only consider as ER that which is carried out according to reason and will. On the contrary, ER that occurs outside of volition should not be called ER. Considering that voluntariness is what defines the concepts of voluntary EM and human ER, it seems appropriate to conclude that both concepts describe the same reality. Therefore, involuntary ER cannot properly be considered ER, unless one wishes to call it so by *analogy*^1^. However, this would be a contradiction in terms: the expression “involuntary emotional regulation” contradicts itself, since ER is always voluntary, as we have argued.

Having said this, we will now move on to answer the first three arguments that seemed to demonstrate that involuntary EM can be considered ER.

First, it is true that involuntary EM fits within the definition of ER provided by Gyurak et al. (2011). However, this definition omits to refer to the voluntariness of ER. Considering that human ER is characterized as voluntary, it is possible that this definition is overly broad. As we showed in Table 4, there are several definitions that consider voluntariness as an essential note of ER. These definitions seem closer to the notion of human ER that we are proposing. From this perspective, involuntary EM cannot be considered ER.

Secondly, it is true that adaptive emotional changes do occur in animals. However, they are very different from those that occur in humans when they self-regulate emotionally. Human ER occurs according to reason and will, which move the cogitative to evaluate affects in a reasonable way, from which new affects arise. In contrast, the emotional change of animals does not occur according to reason and will, since animals lack these powers. Their *estimative* –the animal faculty analog to human cogitative (Aquinas, 1920, I, c. 78, a. 4)– assesses according to rules and ends that are inscribed in animal nature, without it being necessary for the animal to know such rules and ends. Hence, the EM of animals are stereotyped: all lions react in the same way to the same affective stimuli. They are not able to govern their affections freely, in the sense that they cannot oppose them. In contrast, human ER is diverse, and even creative, as human beings differ in their way of reasoning and find their own way of coping with their affects. In this sense, there are two ways of responding. It can be said that animals do not possess ER, in the sense of what we understand as human ER, but rather adaptive EM. Now, on the other hand, it also seems correct to say that animals possess animal ER, in the sense that their ER would be in accordance with their animal nature. From this perspective, it would not be appropriate to homologate it with human emotional regulation, since the latter occurs according to reason and will -which animals lack-, but neither with involuntary EM, since it is a type of regulation inappropriate to human nature. In contrast, animal ER is entirely proportionate to animal nature.

Third, it seems to us true that involuntary EM is not equivalent to a chaotic response. However, that does not automatically make it human ER. As we have shown, involuntary EM can occur in humans, which is distinguished from ER due to the absence of voluntariness, but which is also distinguished from chaos in that it acts according to rules and to certain ends. Therefore, differentiating from chaotic reaction is not enough for us to consider involuntary EM as ER.

### 3.5 Can involuntary emotional modification be classified as emotional dysregulation?

In order to answer this question, we must start by clarifying what emotional dysregulation consists of. The most general answer is offered by Macklem (2008), who states: “the failure to regulate emotion is called dysregulation” (p. 13). From this perspective, any response that departs from emotional regulation could be categorized as emotional dysregulation. A second, more narrowly defined response is offered by Cole et al. (2019): “emotion dysregulation is a pattern of emotion regulation that is interpreted as dysregulated based on the implications for interfering with healthy, competent development” (p. 1192). According to this perspective, a response can only be labeled as emotion dysregulation if it constitutes a pattern that could be considered emotion regulation in a different context.

Which of these two uses is the most appropriate for the concept of ER? The term “dys” means: “inseparable prefix, opposite to *eu*, with notion of hard, bad, unlucky, etc.; destroying the good sense of a word, or increasing its bad sense” (Oxford English Dictionary, 1933). This prefix has been widely used in medicine to name various pathologies. On the other hand, the prefix “dis” has a much broader meaning, as it implies a “privative sense, implying removal, aversion, negation, reversal of action” (Oxford English Dictionary, 1933). In other words: while “dys” is used to refer to a specific sense of negation, namely the spoiling of something, “dis” is used for any form of negation.

If we apply this to our discussion, the word “dysregulation” would mean something more specific than simply the lack or absence of regulation, as suggested by Macklem (2008). Indeed, dysregulation would rather mean “bad” regulation. In that sense, Cole et al.' (2019) definition seems to fit more in this spirit. For them, ED is not simply a lack of regulation, but regulation that has gone bad because of its inappropriate context. In contrast, Macklem (2008) concept could be called “disregulation.”

If we accept that ED is a bad form of ER, then it should be distinguished from problems of ER, as already formulated by Cicchetti et al. (1995). While ED would imply a maladaptive pattern of ER, such as emotion suppression (Beauchaine and Hinshaw, 2008), problems of ER would consist of certain difficulties in the execution of a fully adaptive ER, as when someone manages to calm down, but takes longer than optimal. On the other hand, ED should be distinguished from chaotic responding, which lacks recognizable patterns. This is exemplified by Ford (2013) with respect to dissociation: “from a self-regulation perspective, dissociation is a shift to defensive modes of psychological operations rather than a loss or breakdown” (p. 240).

Taking all this into consideration, involuntary EM would be equivalent to ED. Both involve an emotional change guided by certain rules and in order to achieve certain ends. Moreover, both are maladaptive: while involuntary EM occurs in human beings without the participation of their reason –which is the power with which we can respond appropriately to situations–, ED is an inappropriate response to the context. By establishing this equivalence, both concepts illuminate each other. On the one hand, involuntary EM is revealed as a response inappropriate to the context, and on the other hand, ED is revealed as a phenomenon that occurs outside of reason, following purposes that spring from the sensitive dimension and that may even occur against the individual’s will.

### 3.6 Implications for the distinction between ER and ED

The identification of ER with voluntary EM and of ED with involuntary EM aid us to better illuminate the difference between ER and ED. In comparing the two concepts, it has seemed to us that there are at least seven criteria that could help us clarify the difference between ER and ED. Without claiming to be exhaustive, we have taken these concepts from the literature review and theoretical discussion we have wielded up to this point: degree of consciousness, voluntariness, rule, purpose, cogitative motor, adaptive value, and associated psychic consequences (see Table 6).

**Table 6:** Comparison between emotional regulation and emotional dysregulation.

Comparison criteria	Emotional regulation	Emotional dysregulation
1. Degree of consciousness	Conscious or unconscious.	It is mainly unconscious but can also be conscious.
2. Voluntariness	Voluntary	Involuntary
3. Type of rule	Rule of reason	Rule derived from natural inclinations or learned by experience
4. Purpose	Rational good	Partial sensitive good at the cost of total evil
5. Cogitative motor	The reason	The senses
6. Adaptive value	It is adaptive	It is debatable whether it is adaptive in the short term, but in general terms it is maladaptive.
7. Associated psychological consequences	Health, freedom, order, well-being	Pathology, loss of freedom, disorder, malaise
8. Example	Looking at things with perspective to calm down	Dissociating affects so as not to feel dissatisfaction

With respect to the degree of awareness, ER can be conscious or unconscious, or in other words, explicit or implicit. It can be conscious, as when individuals intend to modulate their affect to better adapt to their context, or it can be unconscious or inadvertent, as when in a situation of discomfort they change their body posture to feel more secure. What both possibilities have in common is voluntariness. ED, on the other hand, usually occurs unconsciously, such as when individuals lose control of their impulses but only others are able to realize it. However, it could also be conscious, as when individuals become aware of the moment in which they are dissociating, although they cannot do anything to stop the process. What is common to both cases is the involuntariness of such operations.

The rule followed by the ER is always that of reason. This is capable of considering concepts and principles universally, so that the purpose of the regulated acts would always be toward the rational good. For example, when individuals who are angry perform a breathing exercise in order not to answer their partner in an offensive way, they do so keeping in mind principles such as: “do not harm” or “it is not good to get angry.” On the other hand, the rule that determines deregulation is that of natural inclinations, specifically those that human beings have in common with the rest of living beings, and also the rules that arise from learning from experience. For example, when a victim of a traffic accident begins to feel rejection and to avoid everything related to motoring. In both cases the purpose of deregulation is the sensible good, that is, the good captured by the senses, which is partial in the sense that it seeks immediate satisfaction without thinking about the total human good. For example, in the case of the accident, the person prefers to avoid means of transportation even if this makes him late for work.

The cogitative faculty, in the case of the ER, is moved by reason. This implies that the movement is initiated by the person himself on the basis of the assessment presented by the higher faculty as convenient, according to an evaluation of the circumstances and with a view to the greater good. In contrast, in the ED the cogitative faculty is moved by the senses, so that it follows natural inclinations and rules derived from experience or nature, apart from rational considerations and the will.

ER, insofar as it is executed with a view to a complete human good, is capable of achieving behavior that is adaptive, but it can even go beyond that. Through ER, behaviors can be performed that conform to higher values, which are even capable of getting people in trouble with their context, for example, when some people choose to do the right thing knowing that it will cause incomprehension and rejection. In contrast, ED does not help to achieve adaptive behavior (D’Agostino et al., 2017). Although it works toward immediate ends, which are in accordance with the basic natural inclinations, it is maladaptive because it fails with respect to the medium and long term ends. It is important to consider that, from a Thomistic perspective, human life consists of much more than the attainment of the inclinations that it shares with the rest of living beings, as we have already explained (Aquinas, 1920, I-II, c.94, a.2). Human beings only reach their fullness to the extent that they live in accordance with reason (Aquinas, 1920, II-II, c. 141, a.1), and life according to reason not only pursues adaptation to the environment, but also life in society, the search for truth, transcendence (Aquinas, 1920, I-II, c.94, a.2) and also for happiness (Aquinas, 1920, I-II, c.1). Therefore, although ED aims at some kind of short-term adaptation, it is incapable of bringing human beings closer to their fullness. The ER, on the other hand, can do so, since its criterion comes from reason.

The psychic consequences are diametrically opposed. The ER would be associated with well-being and psychic health, generating the conditions for the human being to act freely, without the interference of disordered and unmanageable emotions. This would facilitate the adaptive behavior mentioned above. On the other hand, ED would be associated with discomfort and psychic disorder, which hinder human beings from acting according to their self-determination.

According to this comparison, it becomes certain that ER and ED are concepts that refer to different realities. This is no coincidence, since they are realities that are defined by contrast. Many definitions of ED include interference with behavior regulated according to rational ends. In other words, a person cannot be emotionally regulated and dysregulated at the same time. It is true that emotional dysregulation has been defined by some authors as emotional regulation out of context. Although it is true that we can mentally abstract emotional regulation, in real life it always occurs in a given context. However, if that context is not the appropriate one for it to occur, then it does not seem appropriate to conceptualize this mechanism as emotional regulation.

From a Thomistic point of view, this opposition is explained from the nature of the human soul, whose faculties are open to the guidance of reason. The faculties of the sensitive dimension can operate autonomously, without listening to reason, but this is improper of human nature, since it is constituted to be properly governed by its higher faculties. To the extent that reason is able to rule the affections in a habitual way, as occurs in virtue, the human being is self-possessed and can act freely. On the other hand, if affections direct their activity, human beings become alienated, lose control of their actions and are unable to conduct their lives in a humane way. Therefore, the rational management of the affections is not compatible with capitulation to them, at least with respect to the same act.

### 3.7 Is PTSD symptomatology an ER mechanism?

After this long discussion, we are finally in a position to answer this question. We have already stated that PTSD symptomatology can be understood as involuntary EM. In turn, we reviewed that it is not appropriate to consider this type of EM as ER, but rather as ED. Consequently, the symptoms of PTSD (see Table 5) should not be considered an ER mechanism –direct relationship–, but of ED –inverse relationship–. As we have argued, this is not because ED lacks rules and purpose, but because human ER is that which is performed according to human nature, which is rational, and as such occurs with the participation of the will. In contrast, ED occurs outside of reason, even against the will of the individual. Table 7 summarizes the relationship between all the concepts discussed throughout this interdisciplinary dialogue.

**Table 7:** Relationship between ER-ED, voluntary-involuntary EM and explicit-implicit ER.

	Emotional regulation		Emotional dysregulation
**Voluntary or involuntary EM**	Voluntary EM		Involuntary EM
**Adaptivity**	Adaptive		Maladaptive
**Explicit or implicit ER**	Explicit ER	Implicit ER	N/A
**Conscious degree**	Conscious	Unconscious	Unconscious
**Example**	e.g. Someone decides to contain my joy.	e.g. Without thinking about it, someone gets more serious at a funeral.	e.g. Someone dissociates when faced with a problem.

## 4 Discussion

The interdisciplinary approach has allowed us to affirm that PTSD symptomatology should be considered as ED. To achieve this, we have made use of the concepts of voluntary and involuntary EM, which are derived from the Thomistic anthropological view. From here, we have been able to distinguish clearly between emotional regulation and dysregulation as two psychological phenomena that are opposed in many ways, but at the same time are similar in that they constitute patterns of EM that follow certain rules (cf. Tamir, 2015) according to certain purposes.

In this article we have proposed that the voluntariness-involuntariness opposition is different from that of consciousness-unconsciousness. This distinction seems to bring us quite close to the proposal of Braunstein et al. (2017), who states that two dimensions or axes can be distinguished within ER: on the one hand, the distinction between implicit and explicit goals, and on the other, the distinction between controlled and automatic processes of change. Everything seems to indicate that implicit-explicit would be homologous to conscious-unconscious, while controlled-automatic would be equivalent to voluntary-involuntary. From our point of view, the second comparison would be inaccurate, since within voluntary acts we can include acts of control, as when one tries to control laughter at a funeral, but we can also include automatic acts, such as the smile of a kind person. On the other hand, within involuntary acts we can include maladaptive acts of control, such as one who is unable to relax and let go, and automatic acts, such as the development of mental lacunae in a trauma. In the end, voluntariness is not synonymous with control, since even lack of control can be voluntary, as when someone chooses to watch a horror movie. Nor is it synonymous with automaticity, since automatic responses can be voluntarily allowed, as when someone disposes to fall asleep. In that sense, the antinomy “voluntary-involuntary” seems to us more appropriate than “controlled-automatic.” In fact, the concept of “control” does not seem so appropriate to describe ER, since it connotes restriction (Cole et al., 1994; Gratz and Roemer, 2003). Moreover, the opposite concept to controlled is not automatic, but uncontrolled, which is different.

These findings challenge the classic definitions of ER, which were possibly developed with the intention of portraying voluntary EM. Indeed, in reviewing the literature on ER it is clear that researchers are trying to better understand how we voluntarily modify our emotions to better adapt to context. Considering that volition is the distinctive note of voluntary emotional modification, and therefore also of human emotional regulation, it seems to us that this notion should be part of the definition of emotional regulation. As we have shown in Table 4, there are already several definitions that include volition as an essential characteristic of ER. It seems to us relevant that the other definitions be revised or reformulated according to the reflection we have wielded in this article.

Although some authors had already affirmed that ED could be considered a type of ER, in this article we have gone deeper into this affirmation, backing it up with the anthropology of Thomas Aquinas. It is interesting to note that for the Italian thinker there is no problem in admitting finality in the field of involuntary actions. Such a proposal may be shocking to the scientistic view, which usually excludes teleology from its explanatory paradigm. However, teleology has been present since the beginnings of clinical psychology. For example, psychoanalysis assumed that psychic activity is driven by *eros* and *thanatos*, both of which strive for satisfaction, and humanistic theory assumed that the human organism was guided by a force or tendency that drives the individual to self-actualization or self-actualization.

Our proposal has some implications for psychotherapy. The emotional dysregulation of patients can be read in the key of involuntary EM, allowing us to hypothesize that symptomatology implies a certain type of order. In other words, it is not chaos, but a mechanism whose motives are sometimes consciously unknown to us. In a way, this concept is already incorporated in psychotherapy. Many times therapists try to understand the meaning of symptoms, discovering in them intentions unnoticed by the patients. For example, from a psychoanalytic point of view, PTSD symptomatology can be interpreted as secondary gain, and from an Adlerian approach, as a self-sabotage which allows patients to exempt themselves from corroborating their inferiority.

Following our proposal, it is not enough for patients to realize that their symptomatology operates involuntarily under a non-rational logic. It is necessary that they reach voluntary EM, since only this allows the criterion to regulate the affects to come from reason, establishing an order that allows a true human life, achieving an appropriate integration to the context and the reduction of symptoms and flexibility when using it. If Freudian therapy consists of “making the unconscious conscious,” a therapy from our paradigm would imply “regulating the dysregulated” or its equivalent “making the involuntary EM voluntary.” The importance of ER for clinical psychology was emphasized by Cole et al. (1994):

Although clinical theory has not defined emotion dysregulation explicitly, emotion regulation is an implied goal of most treatment models. Understanding emotion patterns and their historical roots, learning to recognize emotions and to express them appropriately, and experiencing problematic emotion patterns in order to modify them are major goals of many therapies. (p. 7)

Interestingly, some therapeutic techniques such as mindfulness and EMDR seem to promote just the opposite view. Mindfulness involves accepting emotions, aiming for a non-judgmental awareness rather than active rational control. On the other hand, EMDR uses the technique of bilateral stimulation, which triggers a process of free association -without reason controlling the thoughts that appear-, during which reprocessing of the trauma occurs. In both cases it seems that the aim is to make the voluntary involuntary. Strictly speaking, however, neither case involves abdicating the rational dimension, since patients must make the choice of focusing attention on the present, letting emotions flow or reprocessing the thoughts that arise while bilateral stimulation is performed. Recall that voluntariness is not synonymous with control (Cole et al., 1994). Although mindfulness and EMDR imply opening oneself to uncontrolled thoughts, this is only possible when the person freely agrees to do so, and this requires choice. In short, regulating emotions rationally and voluntarily is not exactly the same as controlling them, but rather choosing the means that best suit the person’s needs, which in some cases may involve letting them flow. From this point of view, both mindfulness and EMDR are therapeutic techniques that are coherent with our proposal since they promote a rational mastery of people over themselves. Other techniques that can be suggested at a therapeutic level are narrative therapy techniques, grounding and mentalization; all of these techniques promote self-awareness and reflection on one’s own mental and bodily states that lead the person to act voluntarily from reason.

However, it seems important to us to discard the idea that ED is a process without meaning, rule or order. As Cole et al. (1994) state: “Dysregulated does not mean unregulated” (p. 8). Whether it is called ED or involuntary EM, in all cases it is possible to discover an order that follows rules different from those we rationally follow and purposes that the subject has not voluntarily set for himself. It seems important for psychology to approach the understanding of these rules, which escape the logic of reason, without losing sight of the fact that human beings reach their fullness to the extent that they live according to the rules that they impose on themselves through their rationality.

At this point, we can answer some of the questions raised in the introduction. Although some symptoms of PTSD may improperly be considered an emotional regulation mechanism, they should essentially be considered dysregulation. Therefore, it is not necessary to question whether PTSD is a pathology. On the other hand, the distinction between ER and ED has become more sharply demarcated. Despite its possible short-term adaptive value, this symptomatology should not be considered part of normal life and should therefore be addressed by the clinician.

It is certainly important to review the empirical applicability of our proposal. To the extent that we can discover in all PTSD symptomatology some kind of rule or order, then it will be possible to corroborate that all dysregulation hides involuntary EM. It would also be interesting to extend this proposal to other types of symptomatology, and even to other types of involuntary human activity. On the other hand, it would be interesting to corroborate whether all ED is an obstacle to achieve a truly human life.

## 5 Conclusion

ER is a topic of high relevance for psychology and of important application for psychotherapy. Trauma-associated symptomatology has been commonly conceptualized as ED, mainly because it does not seem to help achieve successful adaptation. From our point of view, this symptomatology is appropriately classified as ED, since it would consist of involuntary mechanisms, and therefore, not regulated by reason. In contrast, the emotional regulation proper to human beings would always be voluntary: by their very nature they can only live humanely if they live according to their reason.

The contributions of interdisciplinary reflection have been key to sustain this position with clarity and validity. In particular, the anthropological scheme of Thomas Aquinas has shown great aptitude for dialogue with contemporary psychology. The precision of his concepts, the distinction between the rational and sensitive dimensions -which does not deny the unity of the human psyche- and the dynamic understanding of the faculties have been of great help in this occasion, and in others mentioned in the introduction.

We hope that rethinking PTSD symptomatology will stimulate researchers to continue delving into one of the inaugural mysteries of clinical psychology, in order to reach a synthesis where the contributions of great clinicians can be incorporated together with the progress of theoretical and experimental science. The therapeutic benefits of this advance will undoubtedly be well received by our patients.

## Footnotes

^1^To call something “by analogy” means that it is improperly called because of some kind of similarity to the main exemplar. For example, a toy car is not properly a car, but only by analogy.
